# Retraction Note: Factors associated with induced abortion in Nepal: data from a nationally representative population-based cross-sectional survey

**DOI:** 10.1186/s12978-020-0867-6

**Published:** 2020-01-21

**Authors:** Suresh Mehata, Jamie Menzel, Navaraj Bhattarai, Sharad Kumar Sharma, Mukta Shah, Erin Pearson, Kathryn Andersen

**Affiliations:** 1 Ipas Nepal, Baluwatar, Nepal, Do Cha Marg, Ward No.: 04, Kathmandu, 44600 Nepal; 2 Ipas United States, P.O. Box 9990, Chapel Hill, NC 27515 USA; 3 Ministry of Health, Ram Shah Path, Kathmandu, 44600 Nepal

**Retraction Note: Reprod Health (2019) 16:68**

**https://doi.org/10.1186/s12978-019-0732-7**

The authors have retracted this article [1] because it contains significant conceptual and textual overlap with unpublished work from another group. Suresh Mehata, Jamie Menzel, Erin Pearson and Kathryn Andersen agree with this retraction. Navaraj Bhattarai, Sharad Kumar Sharma and Mukta Shah did not respond to correspondence regarding this retraction.
